# Clinical benefit from EGFR-TKI plus ginsenoside Rg3 in patients with advanced non-small cell lung cancer harboring EGFR active mutation

**DOI:** 10.18632/oncotarget.12059

**Published:** 2016-09-16

**Authors:** Yan Li, Yanmei Wang, Kai Niu, Xiewan Chen, Liqin Xia, Dingxi Lu, Rui Kong, Zhengtang Chen, Yuzhong Duan, Jianguo Sun

**Affiliations:** ^1^ Department of Oncology, Xinqiao Hospital, Third Military Medical University, Chongqing, 400037, China; ^2^ Medical English Department, College of Basic Medicine, Third Military Medical University, Chongqing, 400038, China

**Keywords:** advanced NSCLC, EGFR active mutation, targeted molecular therapy, ginsenoside Rg3, angiogenesis

## Abstract

**Purpose:**

Acquired resistance is a bottleneck that restricts the efficacy of epidermal growth factor receptor-tyrosine kinase inhibitor (EGFR-TKI) for lung cancer. Ginsenoside Rg3 is an antiangiogenic agent which can down-regulate the expressions of vascular endothelial growth factor (VEGF) and EGFR. Combination of EGFR-TKI and ginsenoside Rg3 may be a promising strategy to delay acquired resistance. This retrospective study explored the efficacy and safety of this combined regimen in patients with EGFR mutation and advanced non-small cell lung cancer (NSCLC).

**Results:**

By the deadline of March 31^th^ 2016, the median follow-up period reached 22.9 months. The median PFS was significantly longer in group A than in group B (12.4 months vs 9.9 months, P = 0.017). In addition, ORR was significantly higher in group A than in group B (59.6% vs 41.7%, P = 0.049). The median OS in group A showed no extended tendency compared with that in group B (25.4 months vs 21.4 months, P = 0.258). No significant difference in side effects was found between the two groups.

**Methods:**

A total of 124 patients with advanced NSCLC and EGFR active mutation were collected and analyzed. All of them were treated with first-line EGFR-TKI and divided into two groups. In group A (n=52), patients were administered EGFR-TKI plus ginsenoside Rg3 at standard doses. In group B (n=72), patients received EGFR-TKI alone. Progression-free survival (PFS), overall survival (OS), objective response rate (ORR) and side effects were analyzed.

**Conclusions:**

Ginsenoside Rg3 improves median PFS and ORR of first-line EGFR-TKI treatment in EGFR-mutant advanced NSCLC patients, thus providing a new regimen to delay acquired resistance of EGFR-TKI.

## INTRODUCTION

Lung cancer is the malignancy that causes the highest morbidity and mortality worldwide. The 5-year survival rate for lung cancer is less than 20%, despite progress in chemotherapy, radiotherapy, targeted molecular therapy and immunotherapy [1]. Non-small cell lung cancer (NSCLC) is the most common subtype of lung cancer, occurring at the frequency of about 80% [2]. Epidermal growth factor receptor-tyrosine kinase inhibitor (EGFR-TKI) is currently recommended as the standard first-line treatment for advanced NSCLC patients harboring EGFR active mutation, based on the consistent results of a series of phase III randomized trials (IPASS [3], NEJ002 [4], OPTIMAL [5], ENSURE [6], etc.), which has demonstrated superiority over conventional platinum-based chemotherapy in terms of progression-free survival (PFS) and objective response rate (ORR). However, about 60% of patients unfortunately acquired drug resistance after a favorable response to EGFR-TKI for 9-13 months on average [7]. To overcome the drug resistance, i.e. to further extend median PFS and OS with acceptable toxicity and tolerability, new strategies with biologically synergistic combinations are needed.

Tumor growth and progression depends on angiogenesis. Currently, antiangiogenic agents have become a potential anticancer strategy [8]. Previous studies found that EGFR-TKI plus bevacizumab, a recombinant humanized monoclonal antibody targeting vascular endothelial growth factor (VEGF), is highly effective in improving the outcome of NSCLC patients. For example, JO25567 study revealed 16.0 months of median PFS after using erlotinib combined with bevacizumab as first-line therapy in advanced non-squamous NSCLC patients harboring EGFR active mutation [9]. In a trial of OLCSG 1001, advanced NSCLC patients harboring EGFR active mutation also had a median PFS of 14.4 months after receiving combination of gefitinib and bevacizumab [10]. As known to all, various antiangiogenic agents have been applied to clinical treatment. Thus, whether a combination of the antiangiogenic agents other than bevacizumab with EGFR-TKI could benefit patients remains to be explored. Recent findings have indicated that ginsenoside Rg3, an active ingredient extracted from ginseng leachate, exhibits an anti-cancer effect through suppressing angiogenesis in various tumors [11]. Ginsenoside Rg3 down-regulates VEGF expressions in cancer cells and inhibits angiogenesis by targeting hypoxia-induced multiple signaling pathways [12]. Besides, ginsenoside Rg3 displays antineoplastic properties by inhibiting tumor growth, invasion and metastasis, and inducing apoptosis [13, 14]. On the one hand, ginsenoside Rg3 inhibits lung cancer migration and invasion by down-regulating fucosyltransferase IV (FUT4)-mediated EGFR inactivation and blocking MAPK and NF-κB signaling pathways [15]. On the other hand, ginsenoside Rg3 induces tumor cell apoptosis through decreasing cell surface EGFR [16]. Theoretically, the combination of EGFR-TKI and ginsenoside Rg3 may exert not only a synergistic anti-cancer effect, but also a synchronous inhibitory effect on EGFR and VEGF signaling pathways. However, the clinical benefit from the addition of ginsenoside Rg3 to EGFR-TKI still remains unknown. Therefore, we conducted a retrospective cohort study to explore the efficacy and safety of the therapy combining EGFR-TKI and ginsenoside Rg3 in NSCLC patients harboring EGFR active mutation. This study had been approved by the Ethics Committee of Xinqiao Hospital, Third Military Medical University before being conducted.

## RESULTS

### Patient characteristics

**Figure 1:**
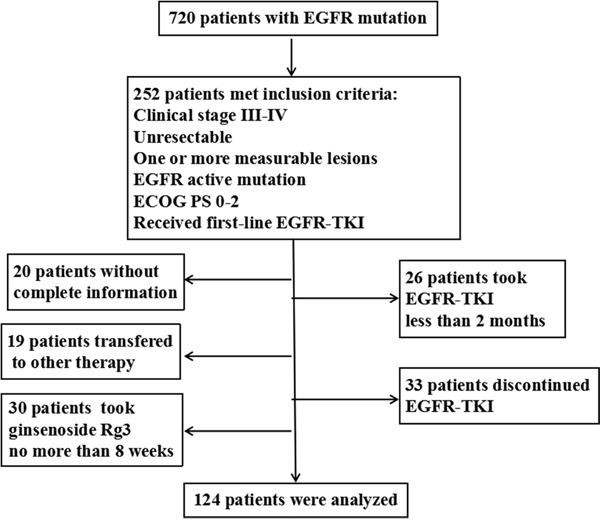
Flow chart of patient selection

A total of 720 patients with EGFR mutation attended the three affiliated hospitals of Third Military Medical University (Xinqiao Hospital, Southwest Hospital and Daping Hospital) from January 2012 to January 2015. Among them, 252 patients met the inclusion criteria. However, 20 patients had no complete information, 30 patients took ginsenoside Rg3 for no more than 8 weeks or uncontinuously, 19 patients adopted other therapies without disease progression, 26 patients were treated with EGFR-TKI for less than 2 months, and 33 patients took EGFR-TKI discontinuously. The remaining 124 patients were collected into the study, aged from 27 to 84 yrs (median age was 58 yrs) (Figure 1). In total, there were 51 males (41.1%) and 73 females (58.9%), and 28 cases were ever or current smokers (22.6%). There were 4, 7 and 113 patients with stage IIIA, IIIB and IV disease respectively. For the 4 stage-IIIA patients, none of them had ever received operation for their rejection to the surgery or other medical reasons. In all the patients, 118 cases were pathologically diagnosed with lung adenocarcinoma (95.2%) and remaining 6 cases were with other subtypes of NSCLC (4.8%). As for EGFR-TKI products, 60 patients received gefitinib, 61 patients erlotinib and 3 patients icotinib.

There were 52 cases in group A and 72 cases in group B, without overlap between groups. All the patients in both groups received first-line EGFR-TKI treatment. And in group A, patients took ginsenoside Rg3 (7.6 ± 6.1 months) continuously during the course of EGFR-TKI. No significant difference existed in gender, age, smoking status, ECOG PS, treatment intervention, distant metastases, clinical stage, histological type, EGFR active mutation sites or treatment line, except for EGFR-TKI products between the two groups. There were 14 cases of erlotinib, 36 cases of gefitinib and 2 cases of icotinib in group A, while 47 cases of erlotinib, 24 cases of gefitinib and 1 cases of icotinib in group B (P < 0.001, Table 1).

**Table 1:** Patient characteristics

Characteristic		Total (n=124)	Group A (n=52)	Group B (n=72)	P value
Gender	Male	51	18	33	0.210
	Female	73	34	39	
Age	≤58	63	28	35	0.565
	>58	61	24	37	
Smoking status	Ever or current	28	10	18	0.448
	Never	96	42	54	
ECOG PS	0	30	10	20	0.490
	1	78	34	44	
	2	16	8	8	
Distant lesions	Bone	70	30	40	0.813
	Brain	43	18	25	0.990
	Liver	16	4	12	0.141
	Adrenal gland	6	3	3	1.000
	Kidney	3	1	2	1.000
Clinical stage	IIIA	4	1	3	0.784
	IIIB	7	3	4	
	IV	113	48	65	
Histological type	Adenocarcinoma	118	49	69	0.682
	Non-adenocarcinoma	6	3	3	
EGFR active mutation sites	Exon 18	1	0	1	0.824
	Exon 19	52	22	30	
	Exon 20	1	0	1	
	Exon 21	43	18	25	
	Unrecorded	27	12	15	
EGFR-TKI	Erlotinib	61	14	47	<0.001
	Gefitinib	60	36	24	
	Icotinib	3	2	1	

### Progression-free survival

**Figure 2:**
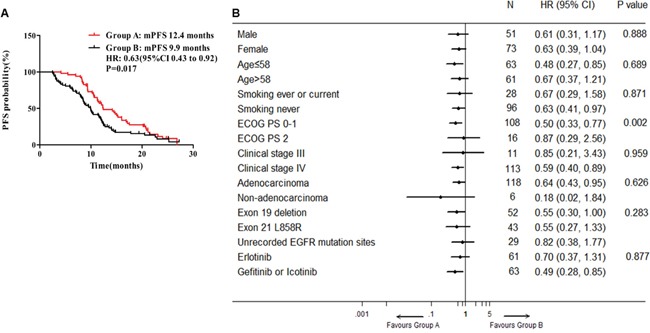
Median PFS and forest plot of hazard ratios for PFS **A.** Median PFS in groups A and B. **B.** Forest plot of baseline characteristics.

By the deadline of March 31^th^ 2016, median follow-up period was 22.9 months. For all the 124 patients, the overall median PFS was 11.2 months (95% CI, 9.9-12.6 months). In groups A and B, median PFS were 12.4 months (95% CI, 9.2-15.6 months) and 9.9 months (95% CI, 8.4-12.6 months) respectively. The median PFS was significantly prolonged in group A compared with group B (Figure 2A, P = 0.017, HR 0.63, 95% CI, 0.43 to 0.92). When subgroup analyses were performed by baseline clinical characteristics in forest plots, most subgroups including age ≤58 (95% CI, 0.27 to 0.85), smoking never (95% CI, 0.41 to 0.97), ECOG PS 0-1 (95% CI, 0.33 to 0.77), clinical stage IV (95% CI, 0.40 to 0.89), adenocarcinoma (95% CI, 0.43 to 0.95) and gefitinib or icotinib (95% CI, 0.28 to 0.85), patients treated with EGFR-TKIs plus ginsenoside Rg3 could benefit from clinical outcome. And subgroup of ECOG PS reaches statistically interactive significance (P = 0.002) (Figure 2B). No matter in groups A or B, the median PFS of EGFR exon 19del were both longer than the ones of EGFR exon 21 L858R, but without significant difference (14.2 months vs 11.6 months, P = 0.809 in group A and 11.5 months vs 8.6 months, P = 0.65 in group B, respectively).

### Overall survival

**Figure 3:**
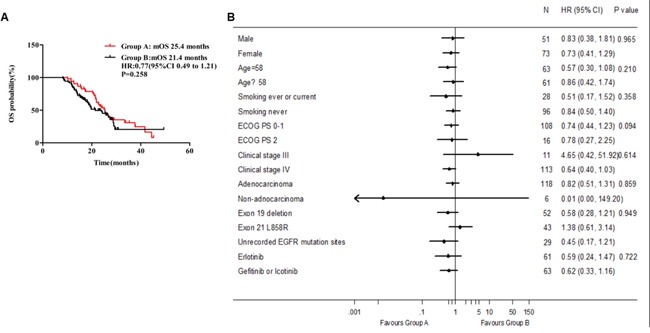
Median OS between group A and group B

By the deadline of follow-up, 34 events (65.4%) had occurred in group A and 42 events (58.3%) in group B. Median OS of 124 patients was 24 months (95% CI, 20.9-27.1 months). The median OS was 25.4 months in group A (95% CI, 22.7-28.0 months) and 21.4 months in group B (95% CI, 15.7-27.1 months). Different from median PFS, ginsenoside Rg3 addition showed no obvious tendency to extend median OS (P = 0.258; HR 0.77, 95% CI 0.49 to 1.21, Figure 3A). There was also no benefit tendency to group A in subgroup OS analysis by all of baseline clinical characteristics (Figure 3B). In groups A and B, 1-year overall survival rate were 94.2% and 84.7% (P = 0.099), and 2-year OS rate were 53.6% and 47.4% (P = 0.129), respectively.

### Response rates

**Figure 4:**
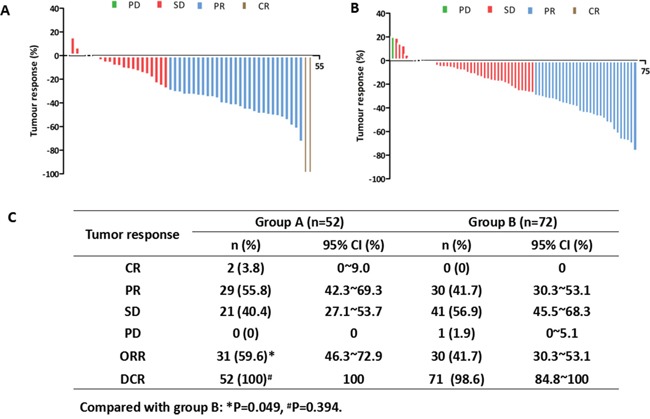
Waterfall plots of best tumor response in groups A and B **A.** Waterfall plot of best tumor response in group A. **B.** Waterfall plot of best tumor response in group B. **C.** Tumor response in groups A and B.

Waterfall plots showed the best tumor response in groups A and B (Figure 4A and 4B). The combination of ginsenoside Rg3 and EGFR-TKI was associated with a higher objective response rate (ORR, CR+PR) in group A compared with group B (59.6% vs 41.7%, P = 0.049), while disease control rate (DCR, CR+PR+SD) reached 100% and 98.6% in group A and group B respectively (P = 0.394, Figure 4C).

### Side effects

Totally, 27 patients (51.9 %) in group A and 39 patients (54.2 %) in group B showed grade ≥ 2 of adverse events and there was no significant difference between the two groups (P > 0.05, Table 2). The most common side effects of any grade in groups A and B were anorexia, nausea and diarrhea. Rash is the only worse side effects in the two groups, 9 patients (17.3 %) in group A and 19 patients (26.4 %) in group B. In addition, the incidences of xerostomia were very low in the two groups. The dry skin and liver function lesion were similar in the two groups. Interstitial pneumonia was reported in 0 patients in group A and 1 (1.4%) patients in group B. No patients in the two groups discontinued erlotinib or ginsenoside Rg3 because of side effects. All patients recovered from above side effects after corresponding treatment.

**Table 2:** Summary of side effects

Characteristics	Group A (n=52) Grade ≤ 2 n (%)	Group B (n=72) Grade ≤ 2 n (%)	P value
Thirsty	2 (3.8)	2 (2.8)	0.233
Dry skin	1 (1.9)	0	0.485
Anorexia	7 (13.5)	7 (9.7)	0.740
Nausea, vomiting	2 (3.8)	0	0.897
Rash	9 (17.3)	19 (26.4)	0.093
Diarrhea	4 (7.7)	6 (8.3)	0.237
Liver function	1 (1.9)	1 (1.4)	0.816
Mouth ulcer	1 (1.9)	3 (4.2)	0.516
Interstitial pneumonia	0	1 (1.4)	0.394

## DISCUSSION

Preclinical studies have indicated that ginsenoside Rg3 is able to exert anticancer effects by inhibiting angiogenesis and tumor growth in many solid tumors such as hepatocellular carcinoma [18], bladder cancer [19], breast cancer [20], ovarian cancer [21] and colorectal cancer [22]. A previous study has also shown the inhibitory effect of ginsenoside Rg3 combined with gemcitabine on the growth of lung cancer in mice [23]. There was also preclinical data showing that ginsenoside Rg3 had the potential of further improving the efficacy of low-dose metronomic temozolomide (TMZ) in the treatment of glioblastoma through addictive antiangiogenic effect [24]. Even ginsenoside Rg3 alone was reported to significantly improve chemotherapy outcomes and extend lifespan of NSCLC patients [25]. A new strategy of EGFR-TKI plus antiangiogenic agents [9, 10] supports a hypothesis that combining EGFR-TKI and ginsenoside Rg3 may benefit advanced NSCLC patients harboring EGFR active mutation. However, no clinical study has reported the effects of ginsenoside Rg3 plus EGFR-TKI in a molecularly defined cohort of advanced NSCLC patients. This retrospective analysis was designed to confirm this speculation in clinical practice.

In the current study, we investigated the clinical effect of the combined therapy based on standard treatment, conventional follow-up visits, general criteria of tumor response and compliance of patients [4, 5, 25]. To the best of our knowledge, this is the first study to determine the efficacy of ginsenoside Rg3 in combination with EGFR-TKI. We found that EGFR-TKI plus ginsenoside Rg3 as the first-line treatment significantly prolonged median PFS in NSCLC patients with active EGFR mutation compared with EGFR-TKI alone. In subgroup analyses, most subgroups showed median PFS benefit from EGFR-TKI plus ginsenoside Rg3. Moreover, the outcome of ECOG PS 0-1 was better than that of PS 2, indicating that patients with good PS status may benefit more from combination of different treatments. Taken together, the results suggest that EGFR-TKI plus ginsenoside Rg3 could be a preferred first-line regimen for advanced NSCLC patients harboring EGFR active mutation. However, whether EGFR-TKI plus ginsenoside Rg3 is also superior to sole EGFR-TKI in second-line treatment remains unknown.

A potential mechanism that may clarify these findings is the effective inhibition of both VEGF and EGFR signaling pathways [26, 27]. Angiogenesis is known to play an important role in growth and metastasis of lung cancer [28]. The most important vascular factors are VEGF and basic fibroblast growth factor (bFGF) [29, 30]. In addition to inhibition of tumor growth signaling, inhibition of VEGF signaling is also essential to overcome drug resistance in tumors harboring EGFR-TKI resistance mutation. In preclinical studies, blocking the VEGF signaling (including bevacizumab and vandetanib) could overcome EGFR-TKI resistance induced by EGFR signaling blockade due to T790M EGFR mutation *in vivo* [31–33].

As an antiangiogenic agent, ginsenoside Rg3 reduces VEGF expressions in cancer cells and attenuates the phosphorylation cascade of the VEGF-dependent p38/ERK signaling *in vitro*, which has also been confirmed in leukemia [12, 34, 35]. Moreover, obviously decreased VEGF expressions in tumor tissues are also found after treatment with ginsenoside Rg3 combined with gemcitabine [23]. Even in hypertrophic scar tissue, ginsenoside Rg3 also exerts an effect of down-regulating VEGF expressions to inhibit fibroblast proliferation [36]. All of the above reports show the analogous effect of ginsenoside Rg3 to bevacizumab. Previous studies found that platinum-based chemotherapy plus bevacizumab was clinically effective and tolerant, and able to prolong median PFS and OS of patients with non-squamous NSCLC [37–39]. Similar results have been reported in treatment that combines ginsenoside Rg3 and platinum-based chemotherapy for NSCLC patients, and ginsenoside Rg3 has been shown to prolong the survival [25]. Consistent with the results of chemotherapy plus bevacizumab, the therapy combining chemotherapy and ginsenoside Rg3 also demonstrates a synergistic effect. In addition, for one thing, ginsenoside Rg3 deactivates the EGFR/MAPK pathway to inhibit tumor cell proliferation [15, 40]. For another, ginsenoside Rg3 reduces cell surface EGFR, and then attenuates EGFR signaling transduction, thus eventually inducing apoptosis in A549 lung adenocarcinoma cells [16].

Considering the analogous antiangiogenic effect of ginsenoside Rg3 to bevacizumab, preclinical studies [31–33] support a hypothesis that ginsenoside Rg3 may have an inhibitory effect on VEGF/EGFR expression when combined with EGFR-TKI. In JO25567 study, EGFR-TKI combined with bevacizumab has been demonstrated to effectively prolong median PFS by inhibiting the VEGF signaling pathway [9]. Accordingly, EGFR-TKI combined with ginsenoside Rg3 can also effectively prolong median PFS of advanced NSCLC patients by a similar mechanism. In our opinion, this antiangiogenic effect of ginsenoside Rg3 analogous to bevacizumab may be the most important factor contributing to the improved median PFS in EGFR-TKI plus ginsenoside Rg3 group. Taking the results of JO25567 [9] and OLCSG 1001 [10] trials together, the current study strengthens a new multidisciplinary strategy to combine EGFR-TKI and antiangiogenic agents as first-line treatment in advanced NSCLC patients harboring EGFR active mutation.

Immunomodulatory effects could be a third mechanism by which ginsenoside Rg3 enhanced the anti-tumor effects of EGFR-TKI. A previous study showed that ginsenoside Rg3 facilitated splenocyte proliferation, induced DC function, and enhanced the carbon clearance from circulating blood [14, 41]. In addition, pharmacological studies proved that ginsenoside Rg3 had other seductive biological properties, such as enhancing anti-fatigue, anti-oxidant, anti-aging and neuroprotective effects, which may also contribute to its antitumor functions [42]. Hence, we believe that the combination of EGFR-TKI and ginsenoside Rg3 has an immunomodulatory effect and is thus able to improve the survival of NSCLC patients. These evidence supports the speculation that combining EGFR-TKI and ginsenoside Rg3 may benefit patients. As our major purpose in this study was to verify the efficacy and safety of EGFR-TKI plus ginsenoside Rg3, the improved median PFS indeed confirmed this speculation clinically.

We also found that the combination of ginsenoside Rg3 and EGFR-TKI was associated with a higher ORR in group A than in group B. These results indicate that ginsenoside Rg3 synergizes with EGFR-TKI treatment, and clinicians may consider the use of EGFR-TKI plus ginsenoside Rg3 to treat advanced NSCLC patients harboring EGFR active mutation.

As for median OS, there was no significant benefit in patients with EGFR-TKI plus ginsenoside Rg3 in our study. This result is possibly due to the limited sample size or the impact of second-line treatment or insufficient administration time of ginsenoside Rg3 (7.6 ± 6.1 months), which needs to be further explored in the future prospective study.

Previous studies have reported that EGFR-TKI has a tendency to be more effective in patients with EGFR exon 19del than in those with exon 21 L858R mutation [5, 43, 44]. In the current study, the median PFS of EGFR exon 19del in group A was longer than that of EGFR exon 21 L858R, but without significant difference (14.2 months vs 10.5 months, P = 0.567). Similar results were observed in group B, in which the median PFS of EGFR exon 19del was longer than that of EGFR exon 21 L858R without significant difference (10.0 months vs 8.6 months, P = 0.841). Additionally, this study displayed that EGFR mutation types preferred to connect to the effect of EGFR-TKI products. Then, it was worth thinking that whether there were relationships between types of EGFR mutation and the effect of ginsenoside Rg3. Besides the data above, the subgroup analysis of median PFS showed that both EGFR exon 19del and exon 21 L858R had a better tendency favoring to group A, but not for unknown or rare mutation. In spite of these, there were no more data on different types of EGFR active mutation, so it was hard for us to draw a conclusion on the association of ginsenoside Rg3 with different types of EGFR. There is also no evidence that the types of EGFR active mutation might influence the effect of another antiangiogenic agent, such as bevacizumab.

As ginsenoside Rg3 and bevacizumab have the commonality that they are both antiangiogenic agents against VEGF. The analogy between the two agents is to better introduce the feasibility of EGFR-TKI combined with antiangiogenesis. However, bevacizumab had been shown to be associated with some special side effects, mainly proteinuria and haemorrhagic events in the studies of JO25567 [9] and OLCSG 1001 [10]. Compared with intravenously administered bevacizumab, ginsenoside Rg3 is administered orally and thus more convenient for patients in clinical use. No hypertension or proteinuria has been found after use of ginsenoside Rg3 in the current and previous studies [23]. No new safety signals were identified and the incidence of side effects (grade ≥2) was similar between the two groups. No obvious increase in toxicity was noted after the addition of ginenoside Rg3. Moreover, ginsenoside Rg3 is generally considered a cheap medicine, costing each patient 250 dollars per month. In contrast, despite significant extension of median PFS by adding bevacizumab to erlotinib [9] or gefitinib [10], the extra bevacizumab charges each patients a price 20 times that of ginsenoside Rg3 per month. Therefore, ginsenoside Rg3 could help improve the therapeutic outcomes of EGFR-TKI without dramatically increasing the total cost of the treatment.

We have also tried our best to control and reduce the probable biases during the current study. All advanced NSCLC patients harboring EGFR active mutation underwent the screening and selection during the research period to minimize the confounding effects of retrospective cohort study. On this basis, our results revealed that the median PFS in patients receiving EGFR-TKI alone was consistent with prior studies [7]. Moreover, the patients in both groups were well matched for demographic and disease characteristics except for EGFR-TKI products. But due to the limitation of sample size and uncontrollability of treatment strategy in retrospective study, there was more use of gefitinib in group A and that of erlotinib in group B. However, the intergroup bias of EGFR-TKI products could not affect the certainty of our conclusion. Previous clinical data showed that erlotinib generated non-shorter median PFS and OS than did gefitinib in first-line treatment and produced longer median PFS and OS than did gefitinib in second-line treatment, and even in some patient subgroups gefitinib was not very effective compared with erlotinib, such as males, non-adenocarcinoma and smokers, which was to say that erlotinib had equal or even prior efficacy in improving patients’ median PFS [4, 7, 45]. Besides, icotinib had similar efficacy to gefitinib [46, 47]. So, in our study, the result with more use of erlotinib in group B but longer median PFS in group A amply proved the contribution of ginsenoside Rg3 to improved median PFS in group A. And a benefit from gefitinib or icotinib subgroup on median PFS (Figure 2B) was also because of the addition use of ginsenoside Rg3 in gefitinib or icotinib subgroup.

Inevitably, there are still several limitations in our study. First, retrospective study is not as powerful as prospective trial. It is difficult to avoid nonrandomized design or distributional difference in EGFR-TKI product selection, which might decrease the credibility of results. Second, a limited sample size is collected. We only included the patients between January 2012 and January 2015 in three hospitals, since that few patients took both EGFR-TKI and ginsenoside Rg3 before 2012 and the observation after 2015 was not long enough to do statistic analyses. Third, there was rare data being collected to further explore the possible relationships between the efficacy of ginsenoside Rg3 and special types of EGFR mutation, such as exon 20 mutation, T790M mutation and so on. Fourth, this study didn't involve the detection of EGFR resistance mutation and molecular changes of cancer tissues. T790M mutation was not a usual examination in hospitals in China during the period of this observation. And, it is hard to obtain cancer tissues to detect relevant signaling pathway by immunohistochemistry (IHC) during the process of EGFR-TKI treatment. Considering the above limitations, we plan to conduct a cohort of patients with prospective randomized design in the near future.

In conclusion, our study provides the first evidence that the addition of ginsenoside Rg3 to EGFR-TKI confers a reduced susceptibility to drug resistance in treating NSCLC patients with EGFR active mutation, and that ginsenoside Rg3 can be a considerable agent to improve patient median PFS and delay acquired resistance to EGFR-TKI. No increased toxicity seems to be found after the addition of ginsenoside Rg3. It also confirms the combination of EGFR-TKI and antiangiogenesis as a new regimen. However, by the limitation of sample size in this study, the definite efficacy and safety of combining ginsenoside Rg3 and EGFR-TKI still warrant further prospective investigation.

## MATERIALS AND METHODS

### Patients

Patients who were histologically or cytologically diagnosed with unresectable stage III or IV advanced NSCLC and attended the three affiliated hospitals of Third Military Medical University (Chongqing, China) between January 2012 and January 2015 were eligible and the medical records of them were retrospectively collected and analyzed. Tumor, node, and metastasis (TNM) classification of lung cancer was based on the 7^th^ edition of the International Association for the Study of Lung Cancer (IASLC) [17]. Inclusion criteria included EGFR active mutations (Exon 18 G719X, Exon 19del, Exon 20 S768I and Exon 21 L858R, etc.), stage III or IV, Eastern Cooperative Oncology Group Performance Status (ECOG PS) 0-2, an age over 18 yrs, first-line and at least two months of EGFR-TKI treatment. Exclusion criteria included incomplete medical record, loss to follow up and less than 8 weeks’ administration of ginsenoside Rg3.

### Treatment

The patients were divided into two groups. In group A, patients were treated with EGFR-TKI plus ginsenoside Rg3 for continuous 8 weeks or longer (according to standard dose of ginsenoside Rg3, 20mg bid, 8 weeks for a course). And in group B, patients received EGFR-TKI alone. EGFR-TKI products (erlotinib, gefitinib and icotinib) were administered at conventional doses (erlotinib 150mg qd, gefitinib 250mg qd, and icotinib 125mg tid). Subsequent chemotherapy drugs after EGFR-TKI failure contained Paclitaxel, Gemcitabine, Docetaxel, Pemetrexed, Cisplatin, Nedaplatin, Carboplatin, etc. And local treatment after local recurrence consisted of palliative surgery, external-beam radiotherapy, interstitial irradiation and thermal therapy.

### Data collection

By the last follow-up on March 31^th^ 2016, the medical records were reviewed to obtain demographic, clinical and pathological information. After 8 weeks’ TKI treatment, tumor response was assessed according to Response Evaluation Criteria in Solid Tumors (RECIST 1.1), including complete response (CR), partial remission (PR), progressive disease (PD) and stable disease (SD). The values of ORR, disease control rate (DCR), median PFS and OS were also analyzed. PFS was defined as the time from the initiating EGFR-TKI treatment to disease progression or death. Likely, OS was defined as the time from the first dose of EGFR-TKI to the death of disease. All patients were followed up at an interval of 1 to 2 months during and after EGFR-TKI treatment, which was also an often follow-up visit [5]. Routine follow-up assessments included physical examinations, vital signs, chest CT, abdomen ultrasound, brain MRI, bone scan (ECT) and laboratory tests. All the side effects including xerostomia, dry skin, insomnia, anorexia, fever, skin rash, joint pain, etc. were monitored and assessed by the National Cancer Institute Common Terminology Criteria for Adverse Events (CTCAE v4.0) during the follow-up.

### Statistical analysis

The ORR, DCR and side effects were compared using the standard Chi-square test or Fisher exact test. The median PFS and OS were estimated using the Kaplan-Meier survival analysis. Differences between groups were compared using the stratified log-rank test. Hazard ratio (HR) and 95% CI were also calculated by Cox proportional hazard regression models. All statistical analyses were performed using SPSS 18.0 software package (SPSS Inc., Chicago, IL, USA). Statistical significance was set at P < 0.05.
